# Delayed post-ischaemic administration of xenon reduces brain damage in a rat model of global ischaemia

**DOI:** 10.1186/cc10912

**Published:** 2012-03-20

**Authors:** V Metaxa, R Lagoudaki, S Meditskou, O Thomareis, A Sakadamis

**Affiliations:** 1 St Bartholomew's Hospital, London, UK; 2 Aristotle University, Thessaloniki, Greece

## Introduction

Cerebral ischaemia is among the leading causes of death, disability and economic expense in the world. Xenon has been shown to be neuroprotective both *in vivo *and *in vitro*, predominantly when administered as a preconditioning agent. We have used a rat model of global ischaemia to investigate whether xenon-induced neuroprotection is observed following an ischaemic insult.

## Methods

Adult male Wistar rats underwent bilateral common carotid artery occlusion and were ventilated for 1 hour with 21% O_2_/78% N_2_. The animals were randomized to receive 21% O_2_/78% N_2_, 50% O_2_/50% N_2_O or 50% O_2_/50% xenon (*n *= 10). After a further 45 minutes, they were killed and their brains were removed for histological, immunochemical and molecular analysis. The numbers of ischaemic neurons in the cortex and the hippocampus as well as the expression of c-fos were evaluated on adjacent brain sections.

## Results

**Figure 1:**
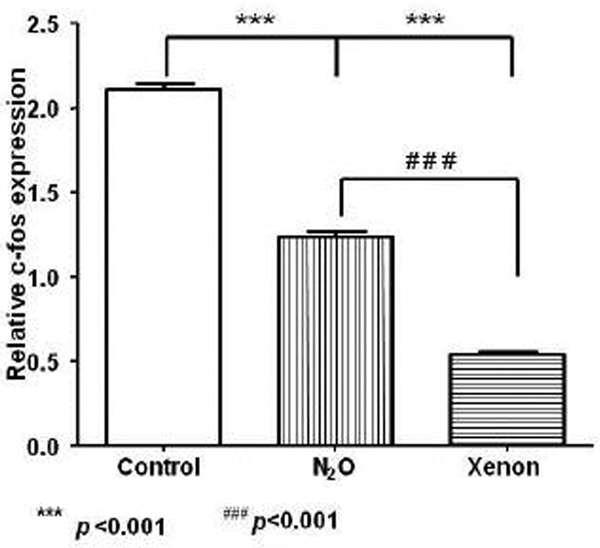
**Regulation of c-fos expression after administration of N_2_O or xenon**.

Both N_2_O and xenon administration reduced the number of ischaemic neurons in the cortex. In xenon-treated rats, fewer ischaemic neurons were also observed in the CA1 region of the hippocampus. The xenon group demonstrated a significant reduction of c-fos expression compared to control and N_2_O groups. See Figure 1.

## Conclusion

In our model of global cerebral ischaemia, the administration of xenon reduced the number of ischaemic neurons compared to control, both in the cerebral cortex and in the hippocampus.
